# Development of a *Ganoderma lucidum* bioreactor for dichlorodiphenyltrichloroethane contaminated soil treatment

**DOI:** 10.1007/s00253-026-13910-1

**Published:** 2026-06-15

**Authors:** Stephanie Casey, Karin Wiberg, Malin Hultberg, Montserrat Sarrà

**Affiliations:** 1 Department of Aquatic Sciences and Assessment, Swedish University of Agricultural Sciences (SLU), SE-750 07 Uppsala, Sweden; 2 Department of Biosystems and Technology, Swedish University of Agricultural Sciences (SLU), SE-234 56 Alnarp, Sweden; 3 Department of Chemical, Biological and Environmental Engineering, Universitat Autònoma de Barcelona, Escola d’Enginyeria, Campus Bellaterra, 08193 Cerdanyola del Vallès, Spain

**Keywords:** Dichlorodiphenyltrichloroethane, Mycoremediation, Contaminated soils, Bioreactor, Surfactant

## Abstract

**Abstract:**

Dichlorodiphenyltrichloroethane (DDT) and its transformation products (collectively referred to as DDX) remain persistent contaminants in Swedish soils despite being banned over 50 years ago. Their partitioning to soil organic matter limits bioavailability and hampers bioremediation efforts. This study created and evaluated a novel pilot bench-scale bioreactor system, which integrates surfactant-assisted soil washing with fungal degradation of DDX for the treatment of aged, contaminated soil. White-rot fungus *Ganoderma lucidum* was investigated for its ability to degrade DDT in a pre-trial, due to its production of high redox potential extracellular ligninolytic enzymes. In a mycelial suspension, *G. lucidum* degraded 73 ± 6% of ∑DDX within 10 days. A prototype trickle-bed bioreactor was then developed in which Tween 80 was continuously circulated through contaminated soil to mobilize DDX into solution before contact with *G. lucidum* colonized woodchips. After 28 days of treatment and a parallel control, ∑DDX in the soil decreased from 10 500 ± 990 µg to 4 300 ± 610 µg in the fungal treatment compared with 7 600 ± 920 µg in the control. Higher mobilization of DDX to the liquid phase was also observed in the treatment bioreactor. Overall, the integrated system reduced ∑DDX by approximately 45% relative to the control and demonstrated effective coupling of soil washing with fungal degradation. These findings highlight the potential of mycoremediation large-scale systems for the treatment of historically contaminated soils.

**Key points:**

- *Bench-scale system integrates washing of aged, contaminated soil with fungal degradation of DDT*
- *Ganoderma lucidum degraded DDT in liquid culture and pilot bioreactor*
- *Surfactant mobilization improved DDT and transformation product availability for fungal treatment*

**Supplementary Information:**

The online version contains supplementary material available at 10.1007/s00253-026-13910-1.

## Introduction

Although banned under the Stockholm Convention (2001), dichlorodiphenyltrichloroethane (DDT) remains a widespread and persistent contaminant in European soils, posing threats to terrestrial and aquatic life through groundwater leaching and bioaccumulation (EFSA 2006). Enhancing its degradation using natural systems is of growing interest, and microbial degradation of DDT has been demonstrated across a range of algae, bacteria, and fungi (Ebsa et al. 2024a). A wide range of fungal species have been investigated for their degradation potential, with different underlying mechanisms being responsible for the degradation. Brown-rot fungi such as *Fomitopsis pinicola* and *Gloeophyllum trabeum* utilize Fenton chemistry for DDT degradation (de Montellano 2018; Purnomo et al. 2011; Rizqi et al. 2023) while white-rot fungi, like *Phanerochaete chrysosporium*, *Pleurotus ostreatus*, and *Trametes versicolor*, have been shown to largely depend on their extracellular ligninolytic enzymes (Mohapatra et al. 2018; Purnomo et al. 2011; Xiao et al. 2011).

The evolutionary role of the ligninolytic enzymes produced by the white-rot fungi is to break down lignin, granting access to otherwise unavailable organic nitrogen sources. These enzymes operate through reactive intermediates, such as hydrogen peroxide and oxalic acid, meaning they can also oxidize a range of anthropogenic compounds, including DDT (Boelan and Purnomo 2018; Kaur et al. 2016). Ligninolytic enzymes generate reactive oxygen species and highly oxidizing radicals, such as veratryl alcohol cation radicals and Mn^3^⁺-chelates, that accumulate in the extracellular environment near fungal cell walls (Hofrichter 2002; Falade et al. 2016). For DDT, the white-rot fungi degradation pathway involves a stepwise transformation including reductive dichlorination to the toxic intermediates DDD and DDE, followed by more water-soluble compounds such as DDA (Aislabie et al. 1997; EFSA, 2006; Mohapatra et al. 2018). After these steps, a wider variety of soil microorganisms continue the degradation to compounds like dichlorobenzophenone (DBP), which is relatively harmless (Wetterauer et al. 2012). Further hydroxylation, oxidation, and ring cleavage eventually lead to mineralization (Bumpus and Aust 1987). DDT and its transformation products are here collectively referred to as DDX.

While fungal treatment has demonstrated promising capability for degradation of DDX in liquid cultures, up to 99% DDX removal (Ebsa et al. 2024b), transferring this to aged, contaminated soils remain a significant challenge. One obstacle is the low bioavailability of these highly hydrophobic pollutants in soils where the DDX remains tightly sorbed to primarily organic carbon but also to mineral surfaces. Still, surfactants can enhance the bioavailability by encapsulating the pollutants within hydrophobic cores of micelles (Guo et al. 2014). Tween 80 is a biodegradable surfactant, non-toxic to fungi or plants (Cheng et al. 2017), which shows minimal inhibition of fungal activity and is promising for use in bioremediation systems (Franzetti et al. 2006), particularly in organic-rich soils. Additionally, some studies have shown that co-culturing fungi with bacteria capable of producing biosurfactants can assist in DDX degradation (Rizqi et al. 2023).

Despite growing interest, affordable, low-impact, and on-site remediation techniques for DDX contaminated soil remain limited. In addition, it is unclear how well fungal systems can perform when coupled with more practical remediation steps, such as surfactant-assisted soil washing.

Therefore, this study prototypes a novel, bench-scale fungal-based soil washing system for DDX degradation combining Tween 80–assisted DDX mobilization from aged soil with a fungal woodchip column. *Ganoderma lucidum*, a white-rot fungus belonging to the order Polyporales, was selected for the study. The production of ligninolytic enzymes like laccases, manganese peroxidase and lignin peroxidase, which are shown to be essential for degradation of pollutants, such as DDT, chlorophenols, and lindane (Deng et al. 2022; Kaur et al. 2016; Ebsa et al. 2024a, b(a); Purnomo et al. 2010), is well-characterized for this species (Zhou et al. 2018; D’Souza et al. 1999). Laccase genes are abundant and highly expressed (CAZy database, Drula et al. 2022), particularly in the pre-fruiting lifestyle phase (Zhou et al. 2018), and the capability of laccases from *G. lucidum* to degrade chlorophenols has been demonstrated (Deng et al. 2022). Additionally, the closely related species *Ganoderma lingzhi* has demonstrated the ability to degrade DDT to DDE and DDD, and possibly further degradation (Boelan et al. 2024; Boelan and Purnomo 2018).

Our study is novel because it applies both soil washing and fungal degradation to aged DDX-contaminated soils from a contaminated site in a single experimental set-up. In the first experiment, a mycelial suspension of *G. lucidum* was studied for its capability to degrade DDX in liquid culture. In the second experiment, aged DDX-contaminated soil was treated in a soil washing process in the developed trickle-bed bioreactor to check for the effectiveness of Tween 80 and *G. lucidum* in an up-scalable, two-stage remediation process to remediate DDT contaminated soils.

## Materials and methods

### Microorganism

*Ganoderma lucidum* FP-58537-Sp was kindly provided by Dr. C.A. Reddy from the Department of Microbiology and Molecular Genetics and the NSF Center for Microbial Ecology, Michigan State University (East Lansing, MI, USA). The fungus was maintained by subculturing every 30 days on malt extract agar plates (2% w/v) at 25 °C. The mycelial suspension was prepared as described previously (Baccar et al. 2011). For production of woodchips colonized by *G. lucidum*, Elm oak (*Quercus ilex*) woodchips were sieved at 4–6 mm and autoclaved as described previously (Beltrán-Flores et al. 2021). Mycelial suspension was used to inoculate the autoclaved woodchips (0.25 L kg^−1^ wood) inside a polyvinyl chloride tray covered with aluminium foil. Culture was incubated at 25 °C during 6 weeks before usage.

### Pure fungi culture degradation of technical DDT by *G. lucidum* (experiment 1)

Mycelial pellets of *G. lucidum* were obtained by inoculating 1 mL of the mycelial suspension in 200 mL malt extract medium (20 g L^−1^) in a 1-L Erlenmeyer flask. The flask was incubated in an orbital shaker (135 rpm) at 25 °C for 5 days. Mycelial pellets were then filtered from the broth and resuspended with a 1:1 v/v ratio of 0.8% NaCl solution, previously to be blended with an X10/20 homogenizer (Ystral GmbH). This suspension was stored at 4 °C until usage, which was within 3 days. The dry weight (dw) of the mycelial suspension (~24 g) was determined by centrifugation at 4000 rpm for 5 min followed by drying at 100 °C for 24 h. The suspension was diluted with 0.8% NaCl solution at a ratio of 1:5 to achieve approximately 4 g dw L^−1^ of mycelium to be used in the experiment.

One treatment and one control were included in the experiment, each performed with three replicates. All flasks contained a total of 50 mL of mycelial suspension. For the control, the mycelial suspension of *G. lucidum* was autoclaved. Technical DDT (product no. DRE-C12080000, LGC) was added to the treatment and control to a final concentration of 2 µM (17.5 µg per 50 mL), based on Huang et al. (2007). Tween 80 was used to solubilize the DDT and was added to the treatments and control in a concentration of 10 mg L^−1^.

### Soil sampling and characterization

Soil cores were collected from the forest nursery at Kolleberga (September 2023, Klippan Municipality, Sweden) to a depth of 20 cm using a soil corer. Following collection, the soil was sieved (2 mm mesh) and stored in darkness at 4 °C. Prior to initiating the experiment, soil was analyzed for DDX as outlined in section “DDX analysis”. Additionally, pH was measured by mixing 1 g of wet soil with 10 mL of deionized water and recorded using a SevenEasy pH meter (Mettler Toledo). Total carbon and nitrogen contents of the soil were determined with a TruMac CN elemental analyzer. The gravimetric water content was obtained by drying the samples overnight at 105 °C. Soil texture was assessed by dry sieving for particles > 2 mm and a combination of wet sieving and PARIO (ISP/Stokes-law) analysis for particles < 2 mm. Soil organic matter content was quantified using loss on ignition (LOI) measurement following combustion at 550 °C for 4 h. The soil characterization data are summarized in Table S1, and analytical methods are further detailed under “Methods description for basic soil characterisation” in the SI.

### Trickle-bed bioreactor treatment with* G. lucidum *(experiment 2)

**Fig. 1:**
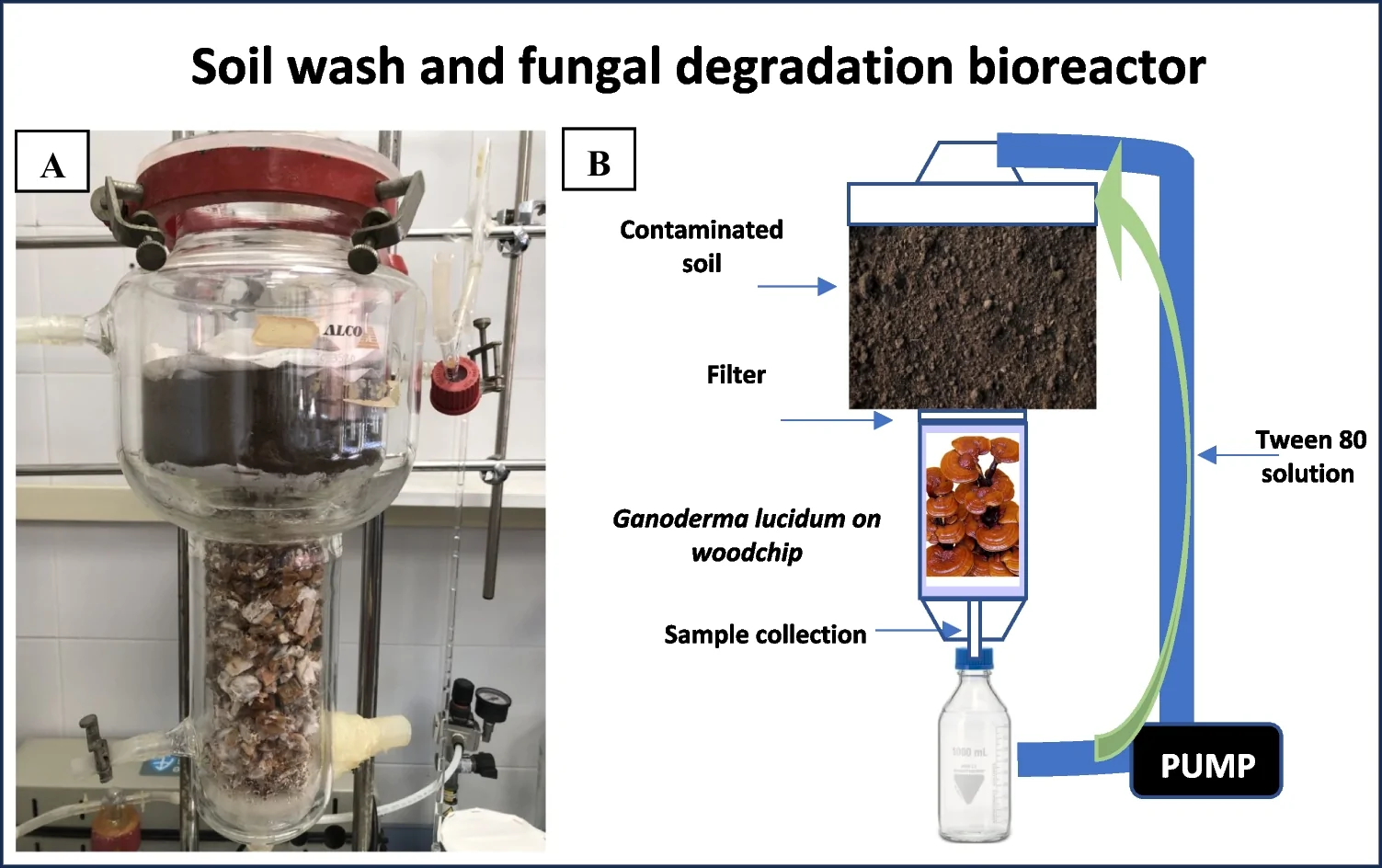
Photo (**A**) and schematic (**B**) of the bioreactor treatment set-up. The control bioreactor was identically assembled but with autoclaved woodchip

Two glass bioreactors, size 2 L, usually operated as airflow fluidized bed bioreactor (Blánquez et al. 2007), are adapted for the experiment as outlined in Fig. 1. The bioreactor comprises two parts: the lower part is a cylindrical of approximately 1 L (diameter 5 cm, height 15 cm) where air is introduced through a holed ceramic plate with pore size under 1 mm, and the upper part with a diameter 20 cm and height 15 cm. The lower chamber was filled with 100 g of woodchips colonized with *G. lucidum* resting on the ceramic plate. The control bioreactor contained 100 g of autoclaved woodchips. A wire mesh filter with a cotton sleeve was placed above the woodchips as a separator. The upper chamber was then filled with 1 kg of contaminated soil. Glass bottles with a solution of 1 L of Tween 80 (Sigma Aldrich, product no. P1754) in a concentration of 1 g L^−1^ were placed under the decanting pipe of the bioreactors. Tween solution was continuously pumped at the rate of 6 mL min^−1^ to the top of the bioreactor through glass and silicon tubing using a peristaltic pump. The bioreactors were allocated inside the laboratory where temperature was maintained at 22–24 °C. Every 3–5 days for 28 days, 50 mL of circulating liquid was collected for DDX analysis and replaced with the same volume of fresh Tween 80 solution. Samples of soil and woodchip were taken at the start and end of the experiment for DDX analysis.

### DDX analysis

Chemicals and materials used, along with their purities and vendors, are detailed in Table S2. Glassware for Soxhlet was burned at 400 °C and rinsed with dichloromethane (DCM) prior to usage. Remaining glassware were burned at 400 °C and rinsed with ethanol before usage. The target analytes were the parent compounds, *p,p*′- and *o,p*′-DDT, and their transformation products *p,p*′- and *o,p*′-DDE (1,1-dichloro-2-bis(p-chlorophenyl)ethene), *p,p*′- and *o,p*′-DDD (1,1-dichloro-2,2-bis(p-chlorophenyl)ethane), *p,p*′-DDM (2,2-bis(chlorophenyl)methane), *p,p*′-DDMU (1-chloro-2,2-bis(p-chlorophenyl)ethene), *p,p*′-DBP (4,4′-dichlorobenzophenone), and dicofol.

#### DDX analysis: extraction and sample preparation of liquid samples

Liquid–liquid extraction was used for analysis of the DDX concentrations in the liquid fraction of experiments 1 and 2. DCM (~20 mL) was combined with the aqueous portion (~ 20 mL) of each sample in a 100 mL separation funnel. To each extraction, 10 µL of an internal standard solution containing 7 ^13^C-labelled DDX compounds (1 ng µL^−1^ each; Table S3) was introduced. The funnel was shaken manually for 1 min and allowed to settle for 5 min until two distinct layers (water and DCM) were observed. The bottom layer (DCM) was carefully collected into a conical flask, and the extraction process was repeated once. To remove residual water from the DCM phase, sodium sulphate (Na₂SO₄) was added to the extract, which was subsequently filtered using 6-µm filter paper (Whatman™ qualitative filter paper, Grade 3, Product number 28413989, Merck KGaA, Darmstadt, Germany) into a round-bottom flask. The solvent volume was reduced via rotary evaporation to approximately 3 mL and transferred to a glass test tube. Further evaporation under a gentle stream of N_2_ followed by solvent exchange to isooctane yielded a final volume of 1 mL. Finally, a recovery standard solution (10 µL, 10 ng mL^−1^) containing ^13^C-PCB 97 and ^13^C-PCB 188 (Table S3) was added before the samples were subjected to GC–MS/MS analysis, detailed in section “DDX analysis: Instrumental analysis by GC–MS/MS.”

#### DDX analysis: extraction and sample preparation of soil and woodchip

After experimental treatment, both soil and woodchip samples were freeze-dried using a LyoDry Compact Benchtop Freeze Dryer (Mechatech Systems, UK) at −55 °C and 7.5E-2 mbar. Subsamples (0.5–1 g) were loaded into pre-cleaned (8 h running of Soxhlet with DCM) Soxhlet thimbles, and 10 µL of a solution containing ^13^C-labelled internal standards (1 ng µL^−1^; Table S3) was spiked into each. The samples underwent Soxhlet extraction using 200 mL of DCM for 24 h following the approach of Huang et al. (2018). Extracts were concentrated by rotary evaporation to 5 mL and then transferred into test tubes. After further evaporation under nitrogen, the solvent was exchanged to *n*-hexane and adjusted to a final volume of 1 mL. A procedural blank containing DCM and internal standards was included for every batch processed.

To remove sulfur, 1 g of activated granular copper was added, and the samples were mixed thoroughly. Further clean-up was performed via column chromatography using 10-mm diameter glass columns packed with 6.1 g of Al₂O₃:SiO₂ (1:2 v/v) and topped with 3 g of Na₂SO₄, following Huang et al. (2018). Target analytes were eluted with 45 mL of a DCM:*n*-hexane (2:3 v/v) solution; the extract was evaporated to 5 mL and then transferred into a glass test tube. A final solvent exchange to isooctane was performed, adjusting the volume to 1 mL. Following the addition of a recovery standard solution (10 µL of 1 ng µL^−1^) (Table S3) and vortexing for 5 min, aliquots were placed into GC vials for instrumental analysis.

#### DDX analysis: instrumental analysis by GC–MS/MS

Instrumental analysis was performed using an Agilent 7890 A gas chromatograph coupled to an Agilent 7010 triple quadrupole mass spectrometer (GC–MS/MS Triple Quad) equipped with an Agilent 7693 autosampler. Injections were made in splitless mode at 275 °C using a Siltek®-deactivated goose neck liner (Restek, USA) to improve sample transfer and minimize compound degradation. Chromatographic separation was achieved using a DB-5 capillary column (60 m × 250 µm i.d. × 0.25 µm film thickness, Agilent Technologies). The oven was programmed as follows: initial temperature of 100 °C (1-min hold), ramped at 15 °C min^−1^ to 250 °C, followed by 10 °C min^−1^ to 280 °C (12-min hold), and finally increased by 20 °C min^−1^ to 300 °C (3-min hold). Helium (He) was used as the carrier gas at a constant flow of 2 mL min^−1^. The mass spectrometer operated under electron ionization (EI) at 70 eV, with the ion source and transfer line temperatures maintained at 300 °C and 310 °C, respectively. The triple quadrupole system was run in multiple reaction monitoring (MRM) mode for enhanced selectivity and sensitivity. Precursor ions were filtered in quadrupole 1 (Q1), then fragmented in Q2 using nitrogen as the collision gas (He as quench gas), and then detected in Q3. Quantification and data processing were conducted using Agilent MassHunter Quantitative Analysis software.

#### Quality assurance and quality control

The limit of detection (LOD) was set to a signal-to-noise (S/N) ratio of ≥ 3, and the limit of quantification (LOQ) was set as the lowest calibration solution that could be reliably quantified by the instrument while fulfilling a S/N criterion of ≥ 10. However, if contamination was present in the blanks, the LOQ was defined as follows:

$$LOQ\:=\:average\;procedural\;blanks\:+\:(10\:\times\:stdev\;procedural\;blanks)$$

In cases when the target analyte was < LOD or < LOQ, the data point was excluded from reporting and replacement values were used for statistical purposes only. Dicofol and *o,p*′-DDE were removed from the statistical analysis of both the solid and liquid phase samples in the bioreactor due to most values being below LOQ both before and after treatment. For the same reason, *p,p*′-DDMU was removed from the analysis of experiment 1. Other data points excluded were *p,p*′-DDT from one of the replicates of wood from the treatment bioreactor, *p,p*′-DDT and *p,p*′-DDD from one replicate of the original starting soil used in the experiment.

A *p,p*′-DDT solution of 250 ng L^−1^ was injected (injection volume 2 µL; injection mass 0.5 ng) separately to monitor in-injector degradation of DDT to DDD during the GC-analysis. The degradation remained below 10% for all samples (Table S4), meeting the threshold accepted as per Foreman and Gates (1997), and, as such, the compounds are reported separately.

Individual DDX data points with recoveries below 50% or exceeding 200% were excluded from data evaluation (Tables S5-S7, S9-S10). Recoveries (all compounds with acceptable recovery data) had mean ± standard deviation of 102% ± 13% for solid samples and 88% ± 16% for liquid samples in the bioreactor experiment (experiment 2) (Table S6) and 103% ± 10% in samples from experiment 1 (Table S5).

In experiment 1, violation of recovery limits resulted in exclusion of multiple data points (Table S7). Replicates were excluded from *p,p*′-DDT in *G. lucidum* day 1 (*n* = 1), *G. lucidum* day 10 (*n* = 2), and control day 1 (*n* = 2) and dicofol in control day 1 (*n* = 1)). In experiment 2, two data points were excluded for *p,p*′-DDT in treatment wood and starting soil, and *p,p'*-DDD in starting soil, resulting in *n* = 2. The data point for control day 28 in the liquid fraction of experiment 2 was entirely removed due to low recovery for all compounds. Dicofol did not reach the recovery limit of 50% in the bioreactor samples. This may be attributed to degradation of dicofol to *p,p*′-DBP during the analysis (Yin et al. 2017). As no separate standard solutions were run to check for potential degradation of dicofol to *p,p*′-DBP, dicofol was excluded from further evaluations in the bioreactor experiment.

### Enzyme analysis

#### Ligninolytic enzyme MBTH-DMAB assay

The ligninolytic activity assay was adapted from Kyaschenko et al. (2017) and conducted on liquid culture subsamples. The colorimetric method utilized the oxidative coupling of 3-methyl-2-benzothiazolinone hydrazone (MBTH) and 3-(dimethylamino)benzoic acid (DMAB), with absorbance measurements at 590 nm recorded every 15 s for 10 min using a SpectraMax Plus 384 microplate reader (Molecular Devices, San Jose, CA, USA). Enzyme activity was expressed as U mL^−1^, calculated from a standard curve prepared with purified MnP (Sigma-Aldrich, product no: 803057; CAS: 114995-15-2).

### Statistical analysis

All statistical analyses were conducted in R Studio (2025.09.2 Build 418) using the packages mgcv, lme4, emmeans, tidyverse, and broom. Statistical significance was assessed at *p* = 0.05.

#### Liquid culture degradation study

DDX concentrations were analyzed using linear mixed-effects models. Fixed effects included treatment, time (day 1 vs. day 10), and their interaction. Replicate flasks were included as a random effect. Post hoc pairwise comparisons of total DDX between day 1 and day 10 were conducted using estimated marginal means (emmeans) with Tukey HSD adjustment for multiple comparisons. Additionally, linear mixed models were fitted for individual compounds to examine compound-specific degradation patterns, with the same fixed and random effect structure, and corresponding post hoc tests were performed.

Enzyme activity measured at days 1, 3, 7, and 10 was analyzed using a linear mixed-effects model to account for repeated measurements taken from the same experimental units over time. Treatment (*G. lucidum* vs control), sampling day, and their interaction were included as fixed effects. Flask identity was included as a random effect. Models were fitted using restricted maximum likelihood (REML) and model assumptions were assessed visually.

#### Bioreactor study

Soil and woodchip samples were analyzed for individual DDX compounds. Multiple subsamples (4) were taken from the same experimental unit (bioreactor) for both treatment and control. Means and standard deviation are reported, but as the replicates are not independent, no comparative statistics were used.

To evaluate the effect of treatment on DDT degradation over time, total DDX concentrations (sum of *p,p*′-DDT, *p,p*′-DDE, *p,p*′-DDD, *p,p*′-DBP, *p,p*′-DDMU, and *p,p*′-DDM) in the circulating liquid were analyzed, and values are reported.

## Results

### Liquid culture DDT degradation study

**Fig. 2:**
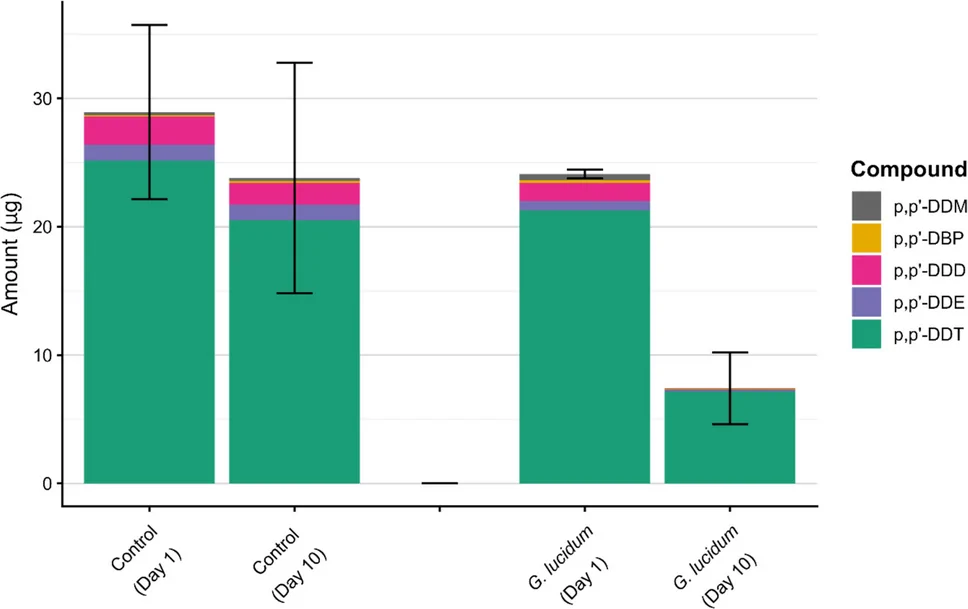
Amounts (µg) of DDX in the liquid cultures pre and post the 10-day incubation period. *n* = 3 except for *p,p*′-DDT in *G. lucidum* day 1 (*n* = 1), *G. lucidum* day 10 (*n* = 2), and control day 1 (*n* = 2), and for dicofol in control day 1 (*n* = 1)

The degradation of DDX in mycelial suspension of *G. lucidum* is shown in Fig. 2, and full data is shown in Table S7. In the control treatment, no significant impact on the ∑DDX concentration was observed when the experiment was ended after 10 days of incubation. In *G. lucidum* treatment, there was a significant decrease in ∑DDX concentration, corresponding to 73 ± 6% of the starting concentration.

**Fig. 3:**
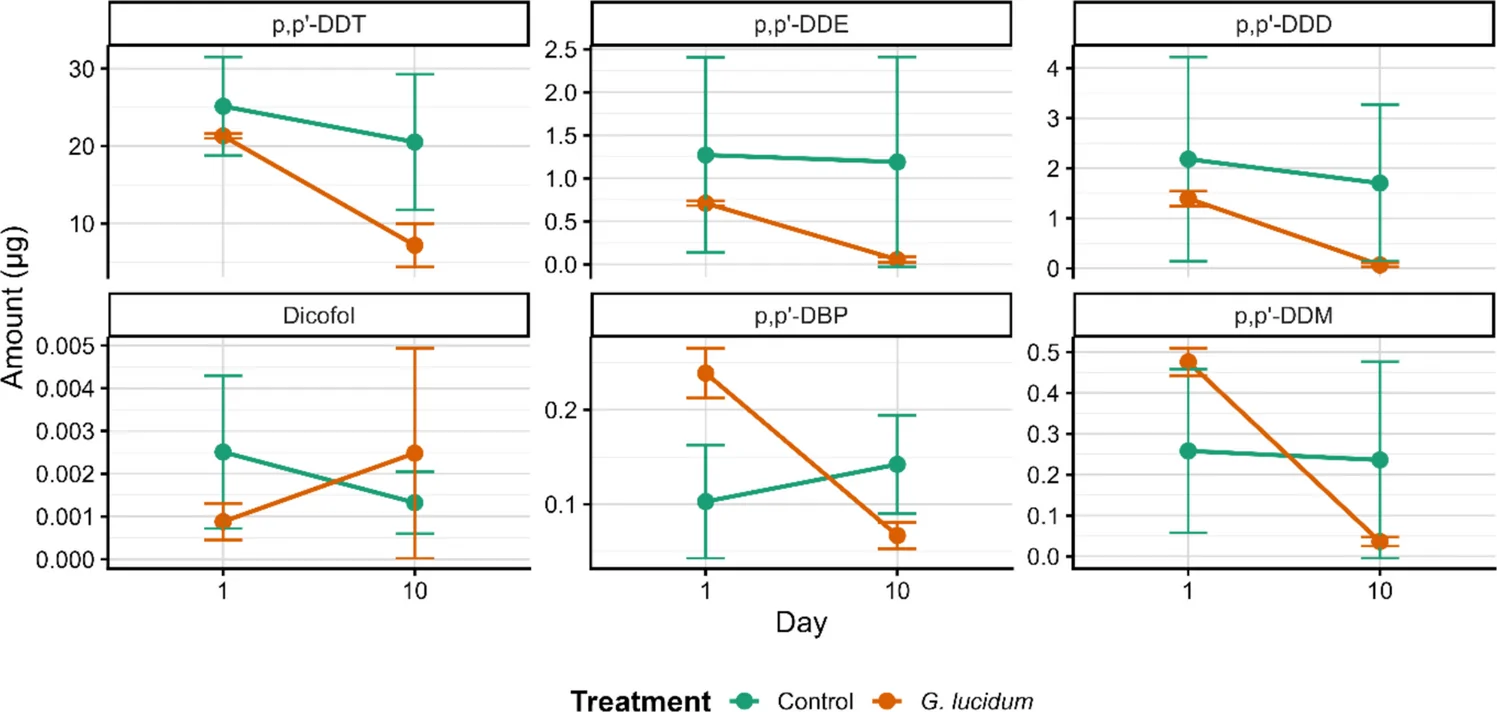
Amounts (µg) of ∑DDX present in the flasks of *G. lucidum* treatment and control before (day 1) and after (day10) treatment with standard deviation error bars. *n* = 3 except for *p,p*′-DDT in *G. lucidum* day 1 (*n* = 1), *G. lucidum* day 10 (*n* = 2), and control day 1 (*n* = 2), and for dicofol in control day 1 (*n* = 1)

No significant decrease in ∑DDX was observed (*p* = 0.30) in the control. In contrast, in the *G. lucidum* culture, the ∑DDX amount fell from 24 to 7.4 µg. The difference was significant, with *p* = 0.018. Due to limited replication, interpretation for individual transformation products is based on estimated effect sizes and confidence intervals rather than formal hypothesis testing, which are shown in full in Table S8. Figure 3 shows that all compounds decreased in the *G. lucidum* treatment except dicofol, and no decreases were observed in the control.

**Fig. 4:**
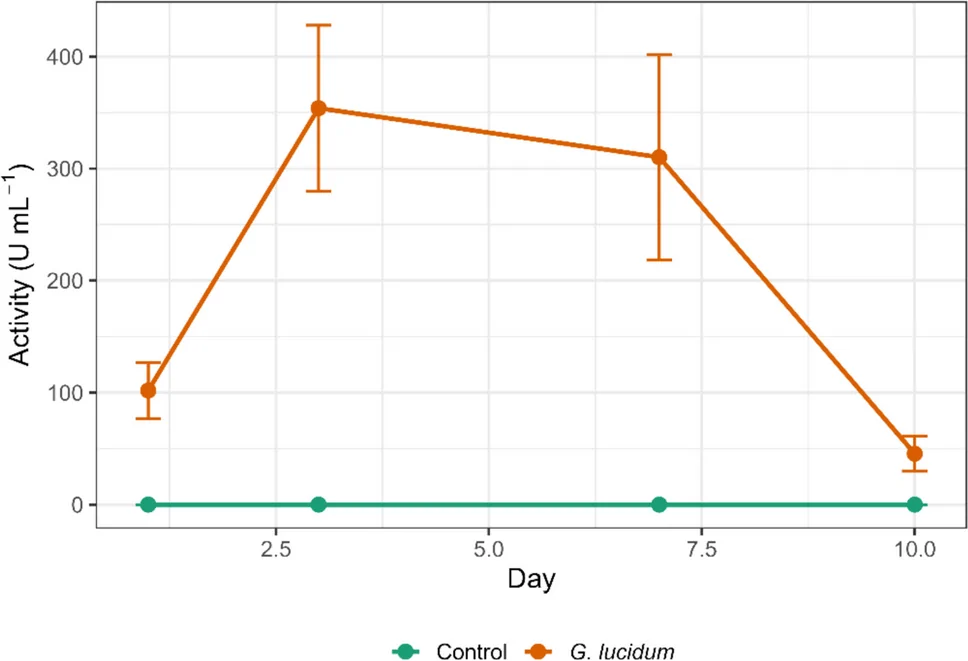
Extracellular ligninolytic enzyme activity (U mL^−1^) over time in the liquid culture experiment (*n* = 3) with standard deviation error bars

The ligninolytic activity detected in fungal treatment was significantly higher than in the control, where no activity was detected (*p* < 0.05) (Fig. 4). Activity appeared to peak earlier in the 10-day period, reaching an observed maximum of 350 U mL^−1^ at day 3. By the end of the experiment, the activity had reduced, considerably indicating that the viability of the fungus had decreased. This experiment demonstrates the capacity of *G. lucidum* to degrade DDX effectively.

### Trickle-bed bioreactor study (experiment 2)

**Fig. 5:**
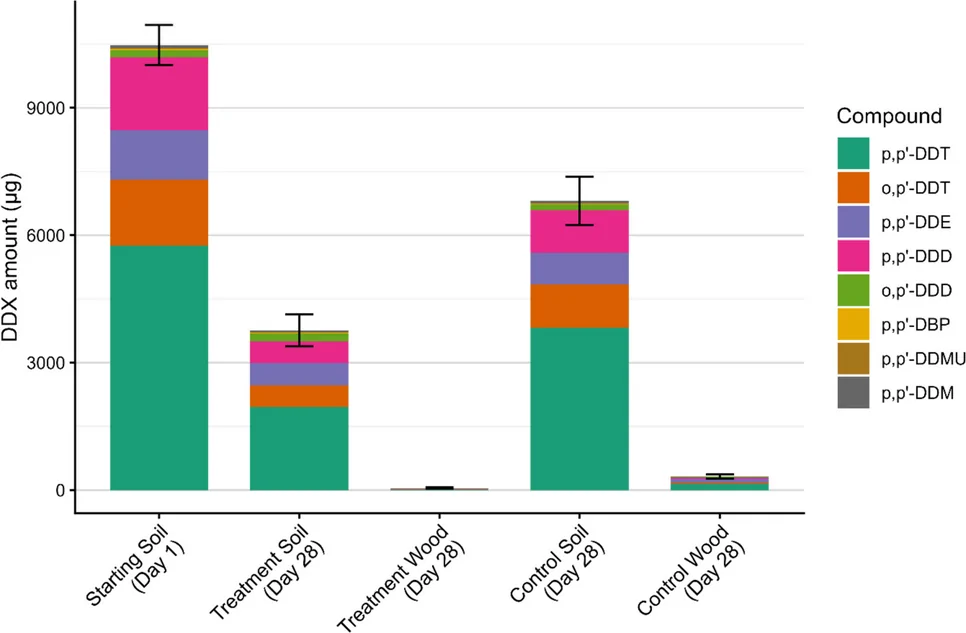
Amounts of DDX (µg) present in the solid phases of the bioreactor at day 1 and day 28 of treatment with standard deviation error bars. *n* = 4 except *p,p*′-DDT in treatment wood and starting soil and for *p,p*′-DDD in starting soil where *n* = 2

The DDX content of the soil and wood matrices are presented in total amounts rather than concentrations in order to avoid considering mass loss changes during the experiment. The calculated total amount of DDX within the soil added to both the control and the treatment bioreactor is shown as “Starting Soil” in Fig. 5. Alongside are the calculated total amounts of DDX in both the woodchip and soil of the control and treatment bioreactors after the 30-day treatment period. All data, averages, and standard deviations are given in Table S9.

The amount of each DDX compound in the soil decreased in both the control and treatment, except for *o,p*′-DDD in the treatment, which appeared unaffected. Treatment reduced the amount of DDX in the soil by approximately 50% compared to the control, likely due to the presence of *G. lucidum*. The starting soil contained 10 500 ± 990 µg total DDX. In the control soil, this was reduced to 7 600 ± 920 µg DDX, while the treatment soil contained 4 300 ± 610 µg DDX. The percentage reduction of each compound between the starting soil (day 1) and the soil samples at day 28 is shown in Table 1. Due to one treatment and one control bioreactor being run, only descriptive statistics are used.

**Table 1:** Percentage reduction of DDX (µg) in soil in relation to the starting soil (day 1) after 28 days of treatment (*n* = 4)

Compound	Average % reduction	
	Treatment soil	Control soil
*p,p'*-DDT	66 ± 5	34 ± 8
*o,p'*-DDT	67 ± 5	35 ± 9
*p,p'*-DDE	54 ± 6	36 ± 7
*p,p'*-DDD	70 ± 4	41 ± 8
*o,p'*-DDD	−9 ± 12	22 ± 10
*p,p'*-DBP	52 ± 12	44 ± 32
*p,p'*-DDMU	44 ± 7	36 ± 10
*p,p'*-DDM	18 ± 2	9 ± 5

**Fig. 6:**
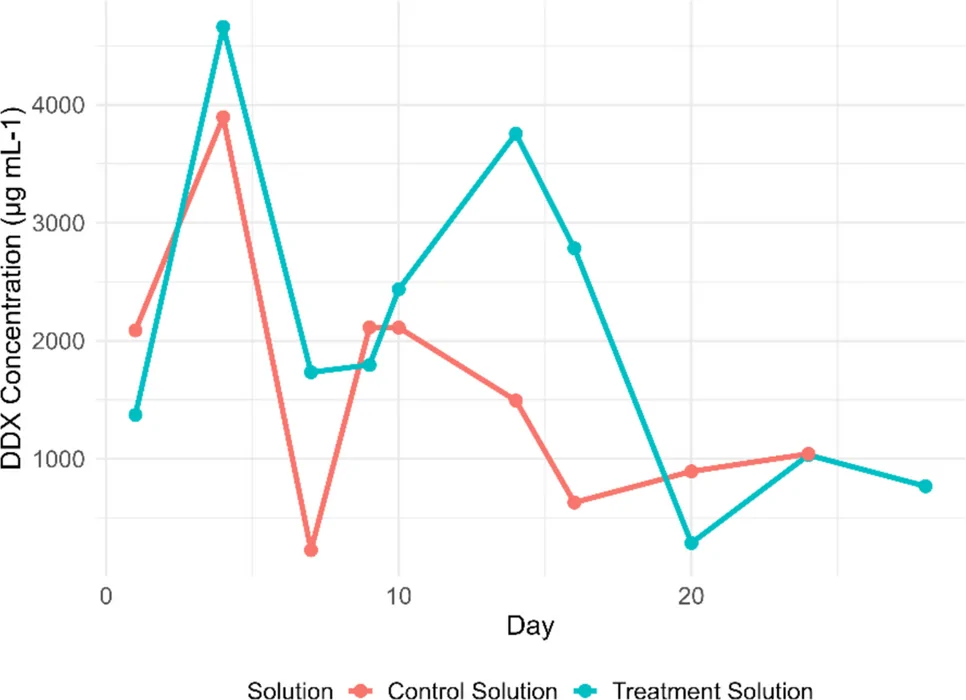
The concentration of DDX (µg mL^−1^) present in the liquid phase of the bioreactor over the 28-day period. The data point related to day 28 of the control reactor was removed due to recovery values for all compounds falling below the acceptable limit

The DDX in the liquid phase of the bioreactor was measured approximately every 3–4 days, and the results are shown in Fig. 6. Similar overall decreasing trends were observed for both treatment and control systems, with the concentration of DDX peaking at the second measurement point, before a sharp decrease at the third. The concentration then increased again for both treatment and control, with the magnitude of DDX mobilization into the liquid phase appearing higher in the treatment with living *G. lucidum*. Due to low power of the study however, this difference cannot be statistically verified. By the end of the experiment, both treatment and control DDX concentrations in the liquid phase had reduced to around 1000 μg mL^−1^.

DDX partitioning among soil, woodchip, and liquid phases was 93%, 1.3%, and 5.5%, respectively, in the treatment, and 90%, 4.9%, and 4.5%, respectively, in the control at the end of the experiment. The absolute amounts at the final day of sampling (solution: day 24; soil and woodchips: day 28) are shown in Table 2.

**Table 2:** Amounts (µg) of ∑DDX in each phase of the bioreactor (experiment 2) at final day of sampling

∑DDX	Control (µg)	Treatment (µg)
Total	8 300	4 600
Soil	7 600	4 300
Woodchips	410	62
Solution	370	250

Three subsamples taken from day 1 and day 28 soils were analyzed for soil organic matter content using LOI analysis. The starting soil contained 3.3% ± 0.3%, while the treatment soil and control soil at day 28 contained 2.6% ± 0.1% and 2.5% ± 0.1%, respectively (Table S11). Here, there was a slight observed reduction in soil organic matter in both the treatment and control bioreactors.

## Discussion

As evident from both Fig. 5 and Table 1, there was a reduction in DDX in the treatment soil, and in the control soil. This reduction may partially be attributed to the native microbial community within the soil, which may possess a limited capacity to degrade certain DDX compounds (Ortíz et al. 2013; Purnomo et al. 2010). This interpretation is supported by the data in Table 1, as the parent compounds and most abundant intermediates (DDT, DDE, and DDD) were reduced more in the treatment than in the control.

Another contributor to the apparent DDX reduction in both the control and the treatment may be the experimental setup itself. The DDX concentrations in the circulating Tween 80 solution are shown in Fig. 6, and the data used are available in Table S10. As seen in the figure, there was an initial loss in DDX concentration between days 4 and 6 for both the control and treatment. A likely explanation for this decrease is that after Tween 80 has mobilized DDX into solution, the compounds initially adsorb onto surfaces within the bioreactor, particularly plastic tubing and fittings. The use of plastic components in the Tween 80 pump system was unavoidable, as was the use of a cotton sheath on the aluminium filter. Hydrophobic compounds, such as DDX, are known to adsorb to these materials as well as, to some extent, to glass surfaces, resulting in potential DDX loss through sorption (Champion and Olsen 1971; Zhang et al. 2021). After the loss phase, increases of DDX concentrations in both control and treatment were seen, potentially caused by material saturation. Alongside the sorption, rapid degradation by the initially abundant ligninolytic enzymes in the inoculated woodchip may also have contributed to the initial rapid decline.

One observed difference between the treatment and control was the amount of DDX released into the liquid phase (Fig. 6). Despite identical Tween 80 circulation, the soil in the control system released slightly less DDX. This may be because the ligninolytic enzymes produced by *G. lucidum* can act on organic matter within the soil, thereby mobilizing sorbed DDX. Furthermore, some fungi, such as *Trametes versicolor*, produce biosurfactants of their own (Lourenço et al. 2017). Though *G. lucidum* has not been tested for this ability, it might be a potential explanation for the higher release of DDX. It should also be pointed out that Tween 80 is known to stimulate ligninolytic enzyme production in white-rot fungi (Ürek and Pazarlioǧlu 2005; Teodoro et al. 2018; Wu et al. 2016), leading to a combined mobilization effect due to increased degradation of organic matter.

Our interpretation of the data is that DDX was released into the liquid phase, and then partitioned between the liquid, soil, and woodchip phases, with losses due to fungal degradation, native microbial activity, and sorption to reactor surfaces. These processes collectively likely explain the reductions in DDX concentrations in the liquid phase and soil at the end of the experiment, and the amount of DDX detected in the woodchips. Similar processes, excluding exposure to white-rot fungal degradation, occurred in the control. Notably, a higher proportion of DDX was sorbed to the woodchips in the control presumably due to the absence of fungal degradation at this site. Furthermore, the compound profile of the bioreactors at day 28 contained a higher proportion of DDT transformation products than the day 10 flasks of the liquid culture degradation study. This indicates that over a longer treatment period, further degradation could have occurred.

Optimization of the bioreactor system could involve adjustment of Tween 80 concentrations, periodic replacement of the surfactant solution, and exploration of alternative fungal growth substrates such as straw. However, the use of Tween 80 may also have unintended effects on soil health. The observed lower organic matter content observed at day 28 compared to day 1 suggests that mobilization of hydrophobic pollutants was accompanied by loss of hydrophobic soil organic matter. Surfactant-based soil washing is reported in literature to cause loss of soil organic matter and fine soil fractions, potentially impairing soil aggregation, water retention, nutrient availability, and soil fertility (Biabani et al. 2023). Previous studies on soil washing have reported reduced soil enzyme activity and microbial diversity due to altered physicochemical properties (Chae et al. 2017; Wang et al. 2020), but this research has been conducted to assess soil washing with acids and has not yet been conducted for Tween 80 surfactant washing. These findings highlight the need to optimize surfactant type and concentration to maximize DDX mobilization while minimizing impacts on soil quality.

Similar systems to the tested one, such as fluidized bioreactors, and packed bed channel bioreactor systems, have all been applied for removal of various hydrophobic pollutants by white-rot fungi, with considerable success (Beltran-Flores et al. 2023; Hu et al. 2022). Tayar et al. (2025) applied a trickle-bed reactor for degradation of two organophosphate flame retardants by *G. lucidum*. Interestingly, this scalable system, which was built for wastewater treatment, was well suited to be used for treating the liquid from washed contaminated soil. Also in that study, *G. lucidum* was inoculated on woodchip and the species establishment and longevity lasted long enough to achieve 85% degradation of two organophosphate flame retardants. Thus, techniques developed for wastewater treatment may be a promising avenue to pursue in future upscale of soil wash and myco-remediation of DDT contaminated soils.

## Conclusion

The novel prototype bioreactor evaluated in this study demonstrates the potential of an integrated system for the treatment of aged DDX-contaminated soils. To our knowledge, this study represents one of the first demonstrations of a prototype reactor integrating surfactant-assisted soil washing with fungal degradation for aged DDX-contaminated field soil. Tween 80 soil washing effectively mobilized DDX from historically contaminated field soils, transferring these compounds into a liquid phase suitable for subsequent fungal degradation by *Ganoderma lucidum.* Importantly, the scale of the present study exceeds that of most previous laboratory investigations of soil washing and fungal remediation of DDX, providing an important step toward practical upscaling. This approach therefore represents a promising alternative to conventional excavation and landfill disposal, enabling excavation with on-site soil washing, soil replacement, and treatment of the contaminated effluent. Overall, these findings highlight the potential of bioremediation systems that incorporate living fungi adapted to engineered treatment environments for the long-term management of DDX contamination.

## Supplementary Information

Below is the link to the electronic supplementary material.

ESM 1 Supplementary Material 1 (DOCX 71.2 KB)

## Data Availability

All data supporting the findings of this study are available within the paper and its Supplementary Information.

## References

[CR1] Aislabie JM, Richards NK, Boul HL (1997) Microbial degradation of DDT and its residues—a review. N Z J Agric Res 40(2):269–282. 10.1080/00288233.1997.9513247

[CR2] Baccar R, Blánquez P, Bouzid J, Feki M, Attiya H, Sarrà M (2011) Decolorization of a tannery dye: from fungal screening to bioreactor application. Biochem Eng J 53(3):184–189. 10.1016/j.bej.2011.06.006

[CR3] Beltrán Flores E, Sarrà M, Blánquez P (2021) Pesticide bioremediation by Trametes versicolor: application in a fixed-bed reactor, sorption contribution and bioregeneration. Sci Total Environ 794:148386. 10.1016/j.scitotenv.2021.148386 34218143 10.1016/j.scitotenv.2021.148386

[CR4] Beltrán Flores E, Pla-Ferriol M, Martínez-Alonso M, Gaju N, Sarrà M, Blánquez P (2023) Fungal treatment of agricultural washing wastewater: comparison between two operational strategies. J Environ Manage 325:116595. 10.1016/j.jenvman.2022.116595 36419290 10.1016/j.jenvman.2022.116595

[CR5] Biabani R, Ferrari P, Vaccari M (2023) Best management practices for minimizing undesired effects of thermal remediation and soil washing on soil properties. A review. Environ Sci Pollut Res 30:103480–103495. 10.1007/s11356-023-29656-6 10.1007/s11356-023-29656-6 37702866

[CR6] Blánquez P, Caminal G, Sarrà M, Vicent T (2007) The effect of HRT on the decolourisation of the Grey Lanaset G textile dye by *Trametes versicolor*. Chem Eng J 126(2–3):163–169. 10.1016/j.cej.2006.09.007

[CR7] Boelan EG, Purnomo AS (2018) Abilities of co-cultures of white-rot fungus Ganoderma lingzhi and bacteria Bacillus subtilis on biodegradation DDT. J Phys: Conf Ser 1095(1):012015. 10.1088/1742-6596/1095/1/012015

[CR8] Boelan EG, Purnomo A, Kamei I (2024) Biotransformation of dichlorodiphenyltrichloroethane by white-rot fungus *Ganoderma lingzhi*. AIP Conf Proc 3071(1):020037. 10.1063/5.0206320

[CR9] Bumpus JA, Aust SD (1987) Biodegradation of DDT [1,1,1-trichloro-2,2-bis(4-chlorophenyl)ethane] by the white rot fungus Phanerochaete chrysosporium. Appl Environ Microbiol 53(9):2001–2008. 10.1128/aem.53.9.2001-2008.1987 3674869 10.1128/aem.53.9.2001-2008.1987 PMC204048

[CR10] Champion DF, Olsen SR (1971) Adsorption of DDT on solid particles. Soil Sci Soc Am J. 10.2136/sssaj1971.03615995003500060015x

[CR11] Chae Y, Cui R, Kim SW, An G, Jeong SW, An YJ (2017) Exoenzyme activity in contaminated soils before and after soil washing: ß-glucosidase activity as a biological indicator of soil health. Ecotoxicol Environ Saf 135:368–374. 10.1016/j.ecoenv.2016.10.007 27771594 10.1016/j.ecoenv.2016.10.007

[CR12] Cheng M, Zeng G, Huang D, Yang C, Lai C, Zhang C, Liu Y (2017) Advantages and challenges of Tween 80 surfactant-enhanced technologies for the remediation of soils contaminated with hydrophobic organic compounds. Chem Eng J 314:98–113. 10.1016/j.cej.2016.12.135

[CR13] de Montellano P (2018) 1-Aminobenzotriazole: a mechanism-based cytochrome P450 inhibitor and probe of cytochrome P450 biology. Med Chem (Los Angeles) 8(3):038. 10.4172/2161-0444.1000495 30221034 10.4172/2161-0444.1000495 PMC6137267

[CR14] Deng W, Zhao W, Yang Y (2022) Degradation and detoxification of chlorophenols with different structure by LAC-4 laccase purified from white-rot fungus *Ganoderma lucidum*. Int J Environ Res Public Health 19(13):8150. 10.3390/ijerph19138150 35805809 10.3390/ijerph19138150 PMC9266351

[CR15] Drula E, Garron ML, Dogan S, Lombard V, Henrissat B, Terrapon N (2022) The carbohydrate-active enzyme database: functions and literature. Nucleic Acids Res 50(D1):D571–D577. 10.1093/nar/gkab1045 34850161 10.1093/nar/gkab1045 PMC8728194

[CR16] D’Souza TM, Merritt CS, Reddy CA (1999) Lignin-modifying enzymes of the white rot Basidiomycete *Ganoderma lucidum*. Appl Environ Microbiol. 10.1128/AEM.65.12.5307-5313.1999 10583981 10.1128/aem.65.12.5307-5313.1999 PMC91721

[CR17] Ebsa G, Gizaw B, Admassie M, Degu T, Alemu T (2024a) The role and mechanisms of microbes in dichlorodiphenyltrichloroethane (DDT) and its residues bioremediation. Biotechnol Rep. 10.1016/j.btre.2024.e00835 10.1016/j.btre.2024.e00835 PMC10972831 38560709

[CR18] Ebsa G, Gizaw B, Admassie M, Desalegn A, Alemu T (2024b) Screening, characterization and optimization of potential dichlorodiphenyl trichloroethane (DDT) degrading fungi. Heliyon. 10.1016/j.heliyon.2024.e33289 39022069 10.1016/j.heliyon.2024.e33289 PMC11253139

[CR19] EFSA Panel on Contaminants in the Food Chain (2006) Opinion of the scientific panel on contaminants in the food chain [CONTAM] related to DDT as an undesirable substance in animal feed. EFSA J 4(12):433–69. 10.2903/j.efsa.2006.433

[CR20] Falade AO, Nwodo UU, Iweriebor BC, Green E, Mabinya LV, Okoh AI (2016) Lignin peroxidase functional applications and its potential in bioremediation. Front Microbiol 7:53527148223

[CR21] Foreman WT, Gates PM (1997) Matrix-enhanced degradation of p,p'-DDT during gas chromatographic analysis: a consideration. Environ Sci Technol 31(3):905–910. 10.1021/es960671q

[CR22] Franzetti A, Di Gennaro P, Bevilacqua A, Papacchini M, Bestetti G (2006) Environmental features of two commercial surfactants widely used in soil remediation. Chemosphere 62(9):1474–1480. 10.1016/j.chemosphere.2005.06.009 16084568 10.1016/j.chemosphere.2005.06.009

[CR23] Guo P, Chen W, Li Y, Chen T, Li L, Wang G (2014) Selection of surfactant in remediation of DDT-contaminated soil by comparison of surfactant effectiveness. Environ Sci Pollut Res 21(2):1370–1379. 10.1007/s11356-013-1993-2 10.1007/s11356-013-1993-2 23900948

[CR24] Hofrichter M (2002) Review: lignin conversion by manganese peroxidase (MnP). Enzyme Microb Technol 0229(01):454–466. 10.1016/S0141-0229(01)00528-2

[CR25] Hu K, Sarrà M, Caminal G (2022) Oak wood provides suitable nutrients for long-term continuous pesticides removal by *Trametes versicolor* in a pilot plant trickle bed reactor. J Clean Prod 380(1):135059. 10.1016/j.jclepro.2022.135059

[CR26] Huang Y, Zhao X, Luan S (2007) Uptake and biodegradation of DDT by 4 ectomycorrhizal fungi. Sci Total Environ 385:1–3. 10.1016/j.scitotenv.2007.04.023 17707073 10.1016/j.scitotenv.2007.04.023

[CR27] Huang H, Zhang Y, Chen W, Chen W, Ding Y, Chen Y, Mao Y, Qi S (2018) Sources and transformation pathways for dichlorodiphenyltrichloroethane (DDT) and metabolites in soils from Northwest Fujian, China. Environ Pollut 2018:560–570. 10.1016/j.envpol.2017.12.071 10.1016/j.envpol.2017.12.071 29329097

[CR28] Kaur H, Kapoor S, Kaur G (2016) Application of ligninolytic potentials of a white-rot fungus *Ganoderma lucidum* for degradation of lindane. Environ Monit Assess 188:588. 10.1007/s10661-016-5606-7 27670886 10.1007/s10661-016-5606-7

[CR29] Kyaschenko J, Clemmensen KE, Hagenbo A, Karltun E, Lindahl BD (2017) Shift in fungal communities and associated enzyme activities along an age gradient of managed *Pinus sylvestris* stands. ISME J 11(4):863–874. 10.1038/ismej.2016.184 28085155 10.1038/ismej.2016.184 PMC5364365

[CR30] Lourenço LA, Alberton Magina MD, Tavares LBB, Ulson G, de Souza SMA, García Román M, Altmajer Vaz D (2017) Biosurfactant production by *Trametes versicolor* grown on two-phase olive mill waste in solid-state fermentation. Environ Technol 39(23):3066–3076. 10.1080/09593330.2017.1374471 28854850 10.1080/09593330.2017.1374471

[CR31] Mohapatra D, Rath SK, Mohapatra PK (2018) Bioremediation of insecticides by white-rot fungi and its environmental relevance. In R. Prasad (Ed.). Mycoremediation and Environmental Sustainabilit, 159–175. 10.1007/978-3-319-77386-5_7

[CR32] Ortíz I, Velasco A, Le Borgne S, Revah S (2013) Biodegradation of DDT by stimulation of indigenous microbial populations in soil with cosubstrates. Biodegradation 24:215–225. 10.1007/s10532-012-9578-1 22847399 10.1007/s10532-012-9578-1

[CR33] Purnomo AS, Mori T, Kamei I, Nishii T, Kondo R (2010) Application of mushroom waste medium from Pleurotus ostreatus for bioremediation of DDT-contaminated soil. Int Biodeterior Biodegradation 64(5):397–402. 10.1016/j.ibiod.2010.04.007

[CR34] Purnomo AS, Mori T, Takagi K, Kondo R (2011) Bioremediation of DDT contaminated soil using brown-rot fungi. Int Biodeterior Biodegradation 65(5):691–695. 10.1016/j.ibiod.2011.04.004

[CR35] Rizqi HD, Purnomo AS, Ulfi A (2023) The effect of bacteria addition on DDT biodegradation by brown-rot fungus *Gloeophyllum trabeum*. Heliyon. 10.1016/j.heliyon.2023.e18216 37519755 10.1016/j.heliyon.2023.e18216 PMC10372667

[CR36] Stockholm Convention (2001) The 12 initial POPs under the Stockholm Convention. https://www.pops.int/TheConvention/ThePOPs/The12InitialPOPs/tabid/296/Default.aspx. Accessed 19 May 2025

[CR37] Tayar S, Villagra J, Gaju N, Martinez-Alonso M, Beltrán Flores E, Sarrà M (2025) *Ganoderma lucidum* immobilized on wood demonstrates high persistence during the removal of OPFRs in a trickle-bed bioreactor. J Fungi 11. 10.3390/jof11020085 10.3390/jof11020085 PMC11856180 39997379

[CR38] Teodoro TS, de Oliveira F, Poffo C, Braga LP, Arbigaus A, Rampinelli JR, Wisbeck E, Bonatti-Chaves M, Furlan SA (2018) The influence of Tween 80 on laccase production by Pleurotus sajor-caju and the efficiency of crude enzyme broth in the removal of bisphenol-A. Arquivos Do Instituto Biológico 85(0). 10.1590/1808-1657001022017

[CR39] Ürek RÖ, Pazarlioǧlu NK (2005) Production and stimulation of manganese peroxidase by immobilized Phanerochaete chrysosporium. Process Biochem 40(1):83–87. 10.1016/j.procbio.2003.11.040 10.1080/10826060701774296 18080906

[CR40] Wang Z, Wang H, Wang H, Li Q, Li Y (2020) Effect of soil washing on heavy metal removal and soil quality: a two-sided coin. Ecotoxicol Environ Saf 203:110981. 10.1016/j.ecoenv.2020.110981 32678759 10.1016/j.ecoenv.2020.110981

[CR41] Wetterauer B, Ricking M, Otte JC, Hallare AV, Rastall A, Erdinger L, Schwarzbauer J, Braunbeck T, Hollert H (2012) Toxicity, dioxin-like activities, and endocrine effects of DDT metabolites-DDA, DDMU, DDMS, and DDCN. Environ Sci Pollut Res 19(2):403–415. 10.1007/s11356-011-0570-9 10.1007/s11356-011-0570-9 21792584

[CR42] Wu M, Xu Y, Ding W, Li Y, Xu H (2016) Mycoremediation of manganese and phenanthrene by *Pleurotus eryngii* mycelium enhanced by Tween 80 and saponin. Appl Microbiol Biotechnol 100(16):7249–7261. 10.1007/s00253-016-7551-3 27102128 10.1007/s00253-016-7551-3

[CR43] Xiao P, Mori T, Kamei I, Kondo R (2011) A novel metabolic pathway for biodegradation of DDT by the white rot fungi, *Phlebia lindtneri* and *Phlebia brevispora*. Biodegradation 22:859–867. 10.1007/s10532-010-9443-z 21184141 10.1007/s10532-010-9443-z

[CR44] Yin G, Athanassiadis I, Bergman Å, Zhou Y, Qiu Y, Asplund L (2017) A refined method for analysis of 4,4′-dicofol and 4,4′-dichlorobenzophenone. Environ Sci Pollut Res 24:13307–13314. 10.1007/s11356-017-8956-y 10.1007/s11356-017-8956-y PMC5434158 28386885

[CR45] Zhang C, Lei Y, Qian J, Qiao Y, Liu J, Li S, Dai L, Sun K, Guo H, Sui G, Jing W (2021) Sorption of organochlorine pesticides on polyethylene microplastics in soil suspension. Ecotoxicol Environ Saf 223:0147. 10.1016/j.ecoenv.2021.112591 10.1016/j.ecoenv.2021.112591 34364123

[CR46] Zhou S, Zhang J, Ma F, Tang C, Tang Q, Zhang X (2018) Investigation of lignocellulolytic enzymes during different growth phases of *Ganoderma lucidum* strain G0119 using genomic, transcriptomic and secretomic analyses. PLoS One 13(5):e0198404. 10.1371/journal.pone.0198404 29852018 10.1371/journal.pone.0198404 PMC5979026

