# Supplementary material for: Development of a Ganoderma lucidum bioreactor for dichlorodiphenyltrichloroethane contaminated soil treatment
Source: Appl Microbiol Biotechnol. 2026 Jun 15;110:188. doi: 10.1007/s00253-026-13910-1 (PMC13294219; doi:10.1007/s00253-026-13910-1)
Supplement: Supplementary file 1 [file 253_2026_13910_MOESM1_ESM.docx]

**Supplementary Information: Development of a *Ganoderma lucidum* bioreactor for dichlorodiphenyltrichloroethane contaminated soil treatment**

S. Casey^a*^, K. Wiberg^a^, M. Hultberg^b^, M. Sarrá^c^

^a^Department of Aquatic Sciences and Assessment, Swedish University of Agricultural Sciences (SLU), SE-750 07 Uppsala, Sweden

^b^Department of Biosystems and Technology, Swedish University of Agricultural Sciences (SLU), SE-234 56 Alnarp, Sweden

^c^Department of Chemical, Biological and Environmental Engineering, Universitat Autònoma de Barcelona, Escola d'Enginyeria, Campus Bellaterra, 08193 Cerdanyola del Vallès, Spain

*Corresponding author: stephanie.casey@slu.se

**Contents**

**Table S1**: Soil characterization data for the Kolleberga forest nursery samples (*n*=3).

**Methods description for basic soil characterisation**

**Table S2**: Details for all chemicals and materials used for chemical analysis

**Table S3**: Target compounds, 13C-labelled internal standards (IS), and 13C-labelled recovery standards (RS), along with vendors, purities, and precursor and product ions.

**Table S4**: Quality control of DDT degradation in the GC-injector. Pre-run meaning after the calibration curve but before the samples, and post run meaning after all samples of that batch have been run.

**Table S5**: DDX recoveries in Experiment 1 (%). Individual DDX data with recovery values outside the recovery limits (<50% or >200%) were excluded from further evaluation. Averages, standard deviations, minimums and maximums are calculated without the excluded values.

**Table S6**: DDX recoveries (%) in the bioreactor experiment (Experiment 2). Values outside the recovery limits (<50% or >200%) were excluded from further evaluation. Averages, standard deviations, minimums and maximums are calculated without the excluded values.

**Table S7**: DDX recoveries (%) in the bioreactor experiment. Individual DDX data with recovery values outside the recovery limits (<50% or >200%) were excluded from further evaluation. Averages, standard deviations, minimums and maximums are calculated without the excluded values.

**Table S8:** Modelled estimated changes (μg) in total DDX and individual DDX compounds over 10 days in control and G. lucidum treatments. Contrasts compare Day 1 vs Day 10 for each treatment. Values shown are the estimated change (estimate), standard error (SE), t-ratio, degrees of freedom (df), and p-value.

**Table S9:** Amounts of DDT and its transformation products (μg) in the original starting soil and in the solid phases of the bioreactors after 28 days of treatment. Individual DDX data with recovery values outside of recovery limits (<50% or >200%), <LOQ or <LOD were excluded from data evaluation (not included in calculations of averages and standard deviation).

**Table S10:** Amounts of DDT and its transformation products (μg) in the liquid phases of the bioreactors at each sampling point during treatment. Values outside of recovery limits (<50% or >200%) were excluded from data evaluation.

**Table S11:** Organic matter (OM) content (%) of bioreactor soils pre- and post- treatment after loss on ignition (LOI).

**Table S1**: Soil characterization data for the Kolleberga forest nursery samples (*n*=3).

| **pH** | **Org-C** | **Clay** | **Silt** | **Sand** | **N-tot** | **C-tot** | **Water content** |
| --- | --- | --- | --- | --- | --- | --- | --- |
|  | % | % | % | % | (g kg^-1^ dw) | (g kg^-1^ dw) | % |
| 6.3 ± 0.3 | 3.1 ± 0.12 | 3 | 16 | 82 | 0.85 ± 0.014 | 15 ± 0.12 | 15±1.5 |

Org-C: organic carbon, N-tot: total nitrogen, C-tot: total carbon

**Methods description for basic soil characterisation**

pH was determined in a 1g wet weight (ww) soil to 10 mL deionized water slurry using a pH meter (S20 SevenEasy pH, Mettler Toledo AB, Stockholm, Sweden). Organic carbon content was measured using loss on ignition (LOI); combustion soil at 505 °C for 4 h. Soil texture was assessed by filtration, and total nitrogen and total carbon were determined by dry combustion (1350 °C) and elemental analysis using a TruMac instrument (LECO Corporation, MI, USA).

**Table S2**: Details for all chemicals and materials used for chemical analysis

| **Product** | **Chemical name** | **Vendor** | **Product number** | **CAS number** |
| --- | --- | --- | --- | --- |
| Calcium carbonate | CaCO_3_ | Sigma-Aldrich | C4830 | 471-34-1 |
| Whatman® cellulose extraction thimbles | - | Merck | WHA10350243 | - |
| Dichloromethane | CH_2_Cl_2_ | VWR International | 1.06054.2500 | 75-09-2 |
| *n*-Hexane | CH_3_(CH_2_)_4_CH_3_ | VWR International | 1.04371.2500 | 110-54-3 |
| Activated granular copper | Cu | Merck | 311405 | 7440-50-8 |
| Aluminum oxide | Al_2_O_3_ | Merck | 544833 | 1344-28-1 |
| Silicon dioxide | SiO_2_ | Merck | 922587 | 7631-86-9 |
| Sodium sulphate | Na_2_SO_4_ | Merck | 238597 | 7757-82-6 |
| Isooctane | (CH_3_)_2_CHCH_2_C(CH_3_)_3_ | Merck | 1.15440 | 540-84-1 |
| 3-Methyl-2-benzothiazolinone-hydrazonehydrochloride (MBTH) | C_8_H_10_ClN_3_S | Merck | 1.04527 | 4338-98-1 |
| 4-(Dimethylamino)-benzaldehyde (DMAB) | C_20_H_27_NO_3_ | Merck | D2004 | 172611-73-3 |
| Manganese-dependant lignin peroxidase (MnP) | - | Merck | 803057 | 114995-15-2 |
| Ethylenediaminetetraacetic acid (EDTA) | (HO_2_CCH_2_)_2_NCH_2_CH_2_N(CH_2_CO_2_H)_2_ | Merck | E6758 | 60-00-4 |
| Hydrogen peroxide solution (30%) | H_2_O_2_ | Merck | 31642-M | 7722-84-1 |
| Sodium acetate | C_2_H_3_NaO_2_ | Merck | 241245 | 127-09-3 |
| Sodium succinate dibasic hexahydrate | NaOOCCH_2_CH_2_COONa · 6H_2_O | Merck | S2378 | 6106-21-4 |
| Sodium DL lactate solution | CH_3_CH(OH)COONa | Merck | L4263 | 72-17-3 |
|  |  |  |  |  |

**Table S3**: Target compounds, ^13^C-labelled internal standards (IS), and ^13^C-labelled recovery standards (RS), along with vendors, purities, and precursor and product ions.

| **Compounds** | **Vendor** | **Chemical purity (%)** | **Precursor ion** | **Product ion** | **Internal standard (IS)** |
| --- | --- | --- | --- | --- | --- |
| **Target compounds** |  |  |  |  |  |
| *p,p′-*DDT | Cambridge Isotope Laboratories, Inc., Massachusetts, USA. | 99.9 | 235 | 165 | ^13^C-*p,p*′*-*DDT |
| *o,p′-*DDT |  | 99.2 | 235 | 165 | ^13^C-*o,p*′*-*DDT |
| *p,p′-*DDE |  | 99.5 | 246 | 176 | ^13^C-*p,p*′*-*DDE |
| *o,p′-*DDE |  | 99.7 | 246 | 176 | ^13^C-*o,p*′*-*DDE |
| *p,p′-*DDD |  | 99.2 | 235 | 165 | ^13^C-*p,p*′*-*DDD |
| *o,p′-*DDD |  | 97.5 | 235 | 165 | ^13^C-*o,p*′*-*DDD |
| *p,p′-*DDMU | Sigma-Aldrich | 98.9 | 212 | 176 | ^13^C-*o,p*′*-*DDE |
| *p,p′-*DDM | LGC Standards, Guilford, UK | 99.5 | 201 | 165 | ^13^C-*o,p*′*-*DDE |
| *p,p′-*DBP | Sigma-Aldrich | 98.7 | 139 | 111 | ^13^C-*o,p*′*-*DDE |
| Dicofol | Cambridge Isotope Laboratories | 99.48 | 251 | 139 | ^13^C*-*dicofol |
|  |  |  |  |  |  |
| **Internal standards (IS)** |  |  |  |  |  |
| 7 DDX solution |  |  |  |  |  |
| ^13^C-*o,p′-*DDT | Cambridge Isotope Laboratories, Inc., Massachusetts, USA. | 99.5 | 247 | 177 |  |
| ^13^C-*p,p′-*DDT |  | >98 | 247 | 177 |  |
| ^13^C-*o,p′-*DDD |  | 96.2 | 247 | 177 |  |
| ^13^C-*p,p′-*DDD |  | 98.2 | 247 | 177 |  |
| ^13^C-*o,p′*-DDE |  | 99.6 | 258 | 188 |  |
| ^13^C-*p,p′-*DDE |  | >98 | 258 | 188 |  |
| ^13^C-Dicofol |  | 99.5 | 263 | 145 |  |
|  |  |  |  |  |  |
| **Recovery standards (RS)** |  |  |  |  |  |
| ^13^C-PCB 97 | Cambridge Isotope Laboratories, Inc., Guelp, Onatorio, Canada. | 99% | 340 | 268 |  |
| ^13^C-PCB 188 |  | 99% | 406 | 336 |  |
|  |  |  |  |  |  |

**Table S4**: Quality control of DDT degradation in the GC-injector. Pre-run meaning after the calibration curve but before the samples, and post run meaning after all samples of that batch have been run.

| GC-MS run | *p,p’*-DDT concentration pre-run  (ng mL^-1^) | *p,p’*-DDD concentration pre-run  (ng mL^-1^) | **Degradation pre-run**  **(%)** | *p,p’*-DDT concentration post-run  (ng mL^-1^) | *p,p’*-DDT concentration post-run  (ng mL^-1^) | **Degradation post-run**  **(%)** |
| --- | --- | --- | --- | --- | --- | --- |
| Bioreactor solid phase | 240 | 31 | **7.7** | 330 | 49 | **6.8** |
| Bioreactor liquid phase | 260 | 33 | **7.9** | 300 | 40 | **7.4** |
| Liquid culture degradation experiment | 240 | 30 | **8.1** | 230 | 30 | **7.7** |

**Table S5**: DDX recoveries in Experiment 1 (%). Individual DDX data with recovery values outside the recovery limits (<50% or >200%) were excluded from further evaluation. Averages, standard deviations, minimums and maximums are calculated without the excluded values.

| **Recovery (%) in experiment 1** | ***p,p'*-DDT** | ***p,p'*-DDE** | ***p,p'*-DDD** | **Dicofol** |
| --- | --- | --- | --- | --- |
| *G. lucidum* -a day 1 | 91 | 89 | 89 | 81 |
| *G. lucidum* -b day 1 | 31* | 91 | 92 | 89 |
| *G. lucidum* -c day 1 | 46* | 78 | 78 | 115 |
| *G. lucidum* -a day 10 | 182 | 97 | 98 | 62 |
| *G. lucidum* -b day 10 | 166 | 100 | 101 | 60 |
| *G. lucidum* -c day 10 | 334* | 86 | 87 | 186 |
| Control-a day 1 | 180 | 101 | 102 | 55 |
| Control-b day 1 | 173 | 102 | 103 | 45* |
| Control-c day 1 | 220* | 97 | 98 | 45* |
| Control-a day 10 | 89 | 98 | 99 | 77 |
| Control-b day 10 | 61 | 97 | 98 | 159 |
| Control-c day 10 | 110 | 99 | 100 | 79 |
| **Average** | 113 | 94 | 95 | 88 |
| **Stdev** | 42 | 19 | 19 | 40 |
| **Min** | 61 | 78 | 78 | 55 |
| **Max** | 182 | 102 | 103 | 186 |

* indicates value excluded due to not fulfilling recovery limit criteria.

**Table S6**: DDX recoveries (%) in the bioreactor experiment. Individual DDX data with recovery values outside the recovery limits (<50% or >200%) were excluded from further evaluation. Averages, standard deviations, minimums and maximums are calculated without the excluded values.

| **Recovery (%) for solid phases in the bioreactor experiment** | ***p,p'*-DDT** | ***o,p*-DDT** | ***p,p'*-DDE** | ***p,p'*-DDD** | ***o,p'*-DDD** |
| --- | --- | --- | --- | --- | --- |
| Treatment Wood-a | 43* | 62 | 87 | 75 | 86 |
| Treatment Wood-b | 56 | 79 | 92 | 96 | 95 |
| Treatment Wood-c | 62 | 73 | 83 | 88 | 84 |
| Treatment Wood-d | 64 | 99 | 130 | 120 | 130 |
| Starting Soil-a | 274* | 192 | 81 | 233* | 89 |
| Starting Soil-b | 196 | 140 | 77 | 170 | 91 |
| Starting Soil-c | 198 | 151 | 82 | 183 | 94 |
| Starting Soil-d | 148 | 115 | 85 | 140 | 93 |
| Control Wood-a | 93 | 80 | 81 | 97 | 92 |
| Control Wood-b | 75 | 72 | 81 | 88 | 89 |
| Control Wood-c | 65 | 66 | 80 | 117 | 83 |
| Control Wood-d | 73 | 69 | 81 | 84 | 89 |
| Treatment Soil-a | 140 | 108 | 82 | 131 | 88 |
| Treatment Soil-b | 140 | 110 | 81 | 133 | 90 |
| Treatment Soil-c | 159 | 124 | 81 | 150 | 86 |
| Treatment Soil-d | 183 | 131 | 82 | 159 | 88 |
| Control Soil-a | 91 | 81 | 81 | 99 | 87 |
| Control Soil-b | 102 | 86 | 80 | 105 | 86 |
| Control Soil-c | 86 | 80 | 83 | 97 | 88 |
| Control Soil-d | 118 | 99 | 85 | 120 | 90 |
| **Average** | 114 | 101 | 85 | 119 | 91 |
| **Stdev** | 47 | 33 | 11 | 30 | 10 |
| **Min** | 56 | 62 | 77 | 75 | 83 |
| **Max** | 198 | 192 | 130 | 183 | 130 |

| **Recovery (%) for liquid phases in the bioreactor experiment** | ***p,p'*-DDT** | ***o,p*-DDT** | ***p,p'*-DDE** | ***p,p'*-DDD** | ***o,p'*-DDD** |
| --- | --- | --- | --- | --- | --- |
| Control day 28 | 15* | 24* | 46* | 38* | 32* |
| Treatment day 24 | 53 | 61 | 62 | 83 | 71 |
| Control day 24 | 61 | 77 | 72 | 91 | 74 |
| Treatment day 24 | 87 | 90 | 69 | 103 | 81 |
| Control day 20 | 55 | 67 | 72 | 109 | 84 |
| Treatment day 20 | 83 | 80 | 55 | 83 | 68 |
| Control day 16 | 60 | 61 | 53 | 102 | 77 |
| Treatment day 16 | 148 | 138 | 69 | 99 | 76 |
| Control day 14 | 53 | 63 | 47* | 89 | 64 |
| Treatment day 14 | 166 | 143 | 54 | 87 | 64 |
| Control day 9 | 124 | 124 | 74 | 106 | 80 |
| Treatment day 9 | 92 | 90 | 60 | 109 | 85 |
| Control day 4 | 189 | 172 | 63 | 104 | 78 |
| Treatment day 4 | 197 | 180 | 52 | 95 | 71 |
| Control day 1 | 84 | 86 | 64 | 112 | 84 |
| Treatment day 1 | 92 | 103 | 84 | 124 | 95 |
| **Average** | 115 | 90 | 102 | 102 | 109 |
| **Stdev** | 42 | 24 | 19 | 19 | 15 |
| **Min** | 46 | 57 | 78 | 78 | 91 |
| **Max** | 182 | 145 | 175 | 176 | 163 |

* indicates value excluded due to not fulfilling recovery limit criteria.

**Table S7:** Amounts of DDT and its transformation products (μg) in Experiment 1 before (T0) and after (T1) treatment. Individual DDX data with recovery values below limit of quantification (<LOQ), below limit of detection (<LOD) or outside the limits of recovery (<50% or >200%) were excluded from further evaluation.

| **Experiment 1 DDX (μg)** | ***p,p'*-DDT** | ***p,p'*-DDE** | ***p,p'*-DDD** | **Dicofol** | ***p,p'*-DBP** | ***p,p'*-DDMU** |
| --- | --- | --- | --- | --- | --- | --- |
| Control day 1-a | 22 | 0.83 | 1.4 | 0.0008 | 0.078 | 0.18 |
| Control day 1-b | 21 | 0.43 | 0.62 | <RL | 0.059 | 0.11 |
| Control day 1-c | <RL | 2.6 | 4.5 | <RL | 0.17 | 0.49 |
| Control day 10-a | 29 | 0.76 | 1.1 | 0.0017 | 0.16 | 0.18 |
| Control day 10-b | 32 | 0.25 | 0.56 | 0.00049 | 0.083 | 0.029 |
| Control day 10-c | 21 | 2.6 | 3.5 | 0.0018 | 0.18 | 0.50 |
| *G. lucidum* day 1-a | 21 | 0.74 | 1.4 | 0.00083 | 0.27 | 0.51 |
| *G. lucidum* day 1-b | <RL | 0.70 | 1.5 | 0.0013 | 0.23 | 0.47 |
| *G. lucidum* day 1-c | <RL | 0.69 | 1.2 | 0.00048 | 0.21 | 0.44 |
| *G. lucidum* day 10-a | 4.4 | 0.029 | 0.032 | 0.00084 | 0.056 | 0.025 |
| *G. lucidum* day 10-b | 7.2 | 0.046 | 0.063 | 0.0053 | 0.082 | 0.047 |
| *G. lucidum* day 10-c | >RL | 0.094 | 0.11 | 0.0013 | 0.062 | 0.036 |

| **Average (μg)** | ***p,p'*-DDT** | ***p,p'*-DDE** | ***p,p'*-DDD** | **Dicofol** | ***p,p'*-DBP** | ***p,p'*-DDMU** |
| --- | --- | --- | --- | --- | --- | --- |
| Control day 1 | 25* | 1.3 | 2.2 | 0.0025* | 0.10 | 0.26 |
| Control day 10 | 21 | 1.2 | 1.7 | 0.0013 | 0.14 | 0.24 |
| *G. lucidum* day 1 | 21* | 0.71 | 1.4 | 0.00088 | 0.24 | 0.48 |
| *G. lucidum* day 10 | 7.2* | 0 | 0 | 0.0025 | 0.067 | 0.036 |
|  |  |  |  |  |  |  |
| **Standard deviation** | ***p,p'*-DDT** | ***p,p'*-DDE** | ***p,p'*-DDD** | **Dicofol** | ***p,p'*-DBP** | ***p,p'*-DDMU** |
| Control day 1 | 5.2 | 0.93 | 1.7 | 0.0015 | 0.049 | 0.16 |
| Control day 10 | 7.1* | 1.0 | 1.3 | 0.00059 | 0.042 | 0.20 |
| *G. lucidum* day 1 | 0.25 | 0.022 | 0.12 | 0.00035 | 0.022 | 0.027 |
| *G. lucidum* day 10 | 2.3 | 0.028 | 0.032 | 0.0020 | 0.011 | 0.0090 |

</>RL indicates removed value from averages and standard deviations calculation due to volitation of recovery limit criteria.

* indicates removed value(s) from the triplicate measurement

**Table S8:** Modelled estimated changes (μg) in total DDX and individual DDX compounds over 10 days in control and *G. lucidum* treatments. Contrasts compare Day 1 vs Day 10 for each treatment. Values shown are the estimated change (estimate), standard error (SE), t-ratio, degrees of freedom (df), and p-value.

| **Compound** | **Sample** | **Estimate (μg)** | **SE** | **t-ratio** | **df** | **p-value** | **Significant** |
| --- | --- | --- | --- | --- | --- | --- | --- |
| Dicofol | Control | 0.001184 | 1.964622 | 0.000603 | 70 | 1 |  |
| Dicofol | *G. lucidum* | -0.0016 | 1.964622 | -0.00082 | 70 | 0.999 |  |
| Total_DDX | *Control* | 5.158047 | 5.589249 | 0.922851 | 10 | 0.378 |  |
| Total_DDX | *G. lucidum* | 16.70896 | 5.589249 | 2.989482 | 10 | 0.0136 | * |
| *p,p'-*DBP | *Control* | -0.03937 | 1.964622 | -0.02004 | 70 | 0.984 |  |
| *p,p'*-DBP | *G. lucidum* | 0.171854 | 1.964622 | 0.087474 | 70 | 0.931 |  |
| *p,p'*-DDD | Control | 0.478408 | 1.964622 | 0.243511 | 70 | 0.808 |  |
| *p,p'-*DDD | *G. lucidum* | 1.327683 | 1.964622 | 0.675796 | 70 | 0.501 |  |
| *p,p'-*DDE | Control | 0.081022 | 1.964622 | 0.041241 | 70 | 0.967 |  |
| *p,p'*-DDE | *G. lucidum* | 0.654768 | 1.964622 | 0.33328 | 70 | 0.74 |  |
| *p,p'*-DDM | Control | 0.022046 | 1.964622 | 0.011222 | 70 | 0.991 |  |
| *p,p'-*DDM | *G. lucidum* | 0.439735 | 1.964622 | 0.223827 | 70 | 0.824 |  |
| *p,p'*-DDT | Control | 4.614759 | 1.964622 | 2.34893 | 70 | 0.0217 | * |
| *p,p'*-DDT | *G. lucidum* | 14.11652 | 1.964622 | 7.185363 | 70 | 5.73E-10 | * |

* indicates significant changes (*p* < 0.05).

**Table S9**: Amounts of DDT and its transformation products (μg) in the original starting soil and in the solid phases of the bioreactors after 28 days of treatment. Individual DDX data with recovery values outside of recovery limits (<50% or >200%), <LOQ or <LOD were excluded from data evaluation (not included in calculations of averages and standard deviation).

| **DDX in solid phases of bioreactor (μg)** | ***p,p'*-DDT** | ***o,p*'-DDT** | ***p,p'*-DDE** | ***p,p'*-DDD** | ***o,p'*-DDD** | ***p,p'*-DBP** | ***p,p'*-DDMU** | ***p,p'*-DDM** |
| --- | --- | --- | --- | --- | --- | --- | --- | --- |
| Starting Soil-a | <RL | 1700 | 1300 | <RL | 95 | 35 | 16 | 38 |
| Starting Soil-b | 5800 | 1800 | 1400 | 1800 | 230 | 35 | 24 | 120 |
| Starting Soil-c | 5600 | 1700 | 1300 | 1700 | 200 | 22 | 22 | 100 |
| Starting Soil-d | <LOQ | 1000 | 770 | <LOD | 130 | 12 | 19 | 55 |
| Treatment Soil-a | 3200 | 890 | 670 | 880 | 130 | 4.3 | 19 | 46 |
| Treatment Soil-b | 3800 | 980 | 740 | 970 | 150 | 22 | 20 | 58 |
| Treatment Soil-c | 3900 | 950 | 700 | 940 | 110 | 8.2 | 17 | 39 |
| Treatment Soil-d | 4500 | 1300 | 880 | 1200 | 140 | 24 | 19 | 56 |
| Treatment Wood-a | <RL | 7.0 | 8.5 | 7.3 | 1.8 | 0.19 | 1.3 | 0.21 |
| Treatment Wood-b | 19 | 5.9 | 7.6 | 6.1 | 2.1 | 0.14 | 1.23 | 0.13 |
| Treatment Wood-c | <LOQ | 17 | 24 | 17 | 3.8 | 0.48 | 1.4 | 0.68 |
| Treatment Wood-d | 12 | 4.8 | 5.3 | 5.0 | 1.2 | 0.080 | 1.2 | 0.10 |
| Control Soil-a | 1900 | 490 | 520 | 490 | 180 | 12 | 17 | 46 |
| Control Soil-b | 2200 | 580 | 590 | 580 | 200 | 11 | 17 | 47 |
| Control Soil-c | 1500 | 400 | 430 | 400 | 150 | 9.3 | 16 | 34 |
| Control Soil-d | 2200 | 570 | 610 | 570 | 200 | 18 | 17 | 47 |
| Control Wood-a | 190 | 47 | 120 | 47 | 34 | 0.91 | 1.9 | 6.8 |
| Control Wood-b | 120 | 31 | 69 | 31 | 18 | 1.2 | 1.6 | 3.6 |
| Control Wood-c | 110 | 28 | 60 | 18 | 16 | 0.32 | 1.6 | 2.8 |
| Control Wood-d | 130 | 33 | 97 | 33 | 24 | 1.5 | 1.8 | 4.9 |
| Solvent Blank-a | 1.2 | 1.1 | 0 | 0.63 | 0.82 | 12 | 0.14 | 1.7 |
| Solvent Blank-b | 1.8 | 0.94 | <LOD | 0.56 | 0.72 | 9.8 | 0.15 | 1.6 |
| Solvent blank-c | 0.67 | 0.52 | <LOD | 0.37 | 0.25 | 8.4 | 0.012 | 1.6 |
| Solvent blank-d | 19 | 3.4 | <LOD | 2.2 | 2.6 | 7.4 | 0.062 | 1.6 |
| **Average** | ***p,p'*-DDT** | ***o,p*'-DDT** | ***p,p'*-DDE** | ***p,p'*-DDD** | ***o,p'*-DDD** | ***p,p'*-DBP** | ***p,p'*-DDMU** | ***p,p'*-DDM** |
| Starting soil (day 1) | 5800* | 1600 | 1200 | 1700* | 170 | 26 | 20 | 78 |
| Treatment Soil (day 28) | 2000 | 510 | 540 | 510 | 180 | 13 | 17 | 44 |
| Treatment Wood (day 28) | 17* | 8.7 | 11 | 8.9 | 2.2 | 0.22 | 1.3 | 0.28 |
| Control Soil (day 28) | 3800 | 1000 | 750 | 1000 | 130 | 15 | 18 | 50 |
| Control Wood (day 28) | 140 | 35 | 87 | 32 | 23 | 0.98 | 1.7 | 4.5 |
| **Standard deviation (μg)** | ***p,p'*-DDT** | ***o,p*'-DDT** | ***p,p'*-DDE** | ***p,p'*-DDD** | ***o,p'*-DDD** | ***p,p'*-DBP** | ***p,p'*-DDMU** | ***p,p'*-DDM** |
| Starting soil (day 1) | 98* | 310 | 230 | 25* | 55 | 9.8 | 3.2 | 32 |
| Treatment Soil (day 28) | 300 | 72 | 69 | 71 | 20 | 3.1 | 0.32 | 5.5 |
| Treatment Wood (day 28) | 3.7* | 4.7 | 7.4 | 4.7 | 1.0 | 0.16 | 0.053 | 0.23 |
| Control Soil (day 28) | 450 | 142 | 79 | 140 | 17 | 8.5 | 1.0 | 8.0 |
| Control Wood (day 28) | 30 | 7.2 | 25 | 10 | 6.9 | 0.44 | 0.13 | 1.5 |

LOQ: Limit of quantification; LOD: Limit of detection; * indicates where *n*=2 or *n*=1 due to removed volitation of quality control criteria.

**Table S10:** Amounts of DDT and its transformation products (μg) in the liquid phases of the bioreactors at each sampling point during treatment. Values outside of recovery limits (<50% or >200%) were excluded from data evaluation.

| **DDX in liquid phase of bioreactor (μg)** | ***p,p'*-DDT** | ***o,p*'-DDT** | ***p,p'*-DDE** | ***p,p'*-DDD** | ***o,p'*-DDD** | ***p,p'*-DBP** | ***p,p'*-DDMU** | ***p,p'*-DDM** |
| --- | --- | --- | --- | --- | --- | --- | --- | --- |
| Treatment day 1 | 45 | 14 | 9.3 | 6.2 | 1.9 | 0.32 | 0.21 | 0.054 |
| Treatment day 4 | 75 | 29 | 33 | 26 | 6.5 | 0.86 | 0.10 | 0.323 |
| Treatment day 7 | 58 | 17 | 120 | 8.3 | 2.4 | 0.43 | 0.22 | 0.061 |
| Treatment day 9 | 46 | 15 | 11 | 6.3 | 1.8 | 0.47 | 0.15 | 0.062 |
| Treatment day 10 | 58 | 20 | 16 | 8.9 | 2.5 | 0.48 | 0.12 | 0.068 |
| Treatment day 14 | 67 | 26 | 26 | 17 | 4.2 | 0.86 | 0.33 | 0.120 |
| Treatment day 16 | 45 | 16 | 14 | 11 | 2.8 | 0.39 | 0.06 | 0.070 |
| Treatment day 20 | 45 | 14 | 11 | 4.1 | 1.3 | 0.43 | 0.14 | 0.024 |
| Treatment day 24 | 39 | 11 | 8.0 | 3.6 | 1.1 | 0.44 | 0.12 | 0.031 |
| Treatment day 28 | 26 | 7.2 | 6.1 | 2.5 | 0.85 | 0.26 | 0.12 | 0.022 |
| Control day 1 | 58 | 19 | 15 | 5.2 | 1.7 | 0.48 | 0.23 | 0.046 |
| Control day 4 | 72 | 27 | 28 | 19 | 5.1 | 0.62 | 0.17 | 0.206 |
| Control day 7 | 4.6 | 0.070 | 0.50 | 0.10 | 4.4 | 0.021 | 0.40 | 0.000 |
| Control day 9 | 49 | 15 | 15 | 12 | 3.2 | 0.44 | 0.22 | 0.070 |
| Control day 10 | 24 | 7.7 | 23 | 32 | 8.9 | 0.62 | 0.20 | 0.036 |
| Control day 14 | 33 | 10 | <RL | 20 | 6.2 | 0.54 | 0.50 | 0.033 |
| Control day 16 | 33 | 0.71 | 6.1 | 13 | 3.4 | 0.30 | 0.23 | 0.023 |
| Control day 20 | 7.52 | 2.0 | 13 | 17 | 5.3 | 0.40 | 0.22 | 0.022 |
| Control day 24 | 17 | 4.0 | 8.3 | 12 | 4.0 | 0.88 | 0.14 | 0.031 |
| Control day 28 | <RL | <RL | <RL | <RL | <RL | <RL | <RL | <RL |
| Solvent blank | 0.50 | 0.57 | <LOD | 0.28 | 0.14 | 6.7 | 0.18 | 1.6 |
| Solvent blank | 0.56 | 0.56 | <LOD | 0.27 | 0.40 | 6.4 | 0.17 | 1.5 |
| Solvent blank | 1.0 | 0.60 | <LOD | 0.31 | 0.46 | 6.9 | 0.16 | 1.5 |

LOD: Limit of detection; RL: Recovery limit

**Table S11:** Organic matter (OM) content (%) of bioreactor soils pre- and post- treatment after LOI.

| **Sample** | **OM(%)** |
| --- | --- |
| Starting soil-a | 3.43 |
| Starting soil-b | 3.52 |
| Starting soil-c | 2.90 |
| Treatment soil-a | 2.54 |
| Treatment soil-b | 2.35 |
| Treatment soil-c | 2.47 |
| Control soil-a | 2.66 |
| Control soil-b | 2.60 |
| Control soil-c | 2.45 |
